# Prevalence and morphology study of distolingual root in mandibular first and second molars in Thai population: a cone-beam computed tomography analysis

**DOI:** 10.1186/s12903-025-06018-x

**Published:** 2025-04-28

**Authors:** Sirintra Chungchatupornchai, Raweewan Arayasantiparb, Titalee Jirathanyanatt

**Affiliations:** 1 Department of Operative Dentistry and Endodontics, Faculty of Dentistry, Mahidol University, 6 Yothi St., Rajthevi, Payathai, Bangkok, 10400 Thailand; 2 Department of Oral and Maxillofacial Radiology, Faculty of Dentistry, Mahidol University, 6 Yothi St., Rajthevi, Payathai, Bangkok, 10400 Thailand

**Keywords:** Distolingual root, Radix entomolaris, Mandibular molars, Root canal morphology, Cone-beam computed tomography

## Abstract

**Background:**

To investigate the prevalence and morphological characteristics of distolingual roots in permanent mandibular molars among a Thai population using a cone-beam computed tomography (CBCT).

**Methods:**

This retrospective study consisted of two parts: prevalence and morphology study. A total of 5228 permanent mandibular first and second molars from 2623 Thai patients were included in the prevalence study, and CBCT images were analyzed to identify the presence of distolingual roots. Additionally, 288 teeth with distolingual roots were subjected to further analysis to assess their root morphology, canal configuration, and orifice location. Statistical analyses were performed using descriptive statistics, Pearson’s chi-square test, paired t-test, and other appropriate methods.

**Results:**

The prevalence of distolingual roots in permanent mandibular molars was 9.14% among the examined teeth and 14.94% among the examined patients. The majority of distolingual roots were classified as type C according to Carlsen and Alexandersen’s classification, and type III according to Song’s classification. Additionally, the vast majority of teeth exhibited Vertucci type I canal configuration. Further analysis revealed that the mean interorifice distances between DB-DL, ML-DL, and MB-ML orifices were 3.01 ± 0.6 mm, 3.43 ± 0.57 mm, and 2.61 ± 0.59 mm, respectively. The angle and radius of curvature of distolingual roots in the proximal view were greater than those in the clinical view (*p* < 0.001).

**Conclusions:**

This study provides comprehensive insights into the prevalence and morphological characteristics of distolingual roots in permanent mandibular molars among a Thai population. These findings emphasize the importance of thorough preoperative assessment and awareness of root morphology variations to enhance the success of endodontic treatment.

## Background

The main objective of endodontic treatment is to eliminate or prevent apical periodontitis. To achieve this goal, the entire root canal system must be thoroughly disinfected and sealed. Untreated root canal tends to be one of the most significant factors contributing to endodontic failure [1]. Awareness and understanding of the anatomy of the tooth and its root canal complex are key factors in enhancing the success of endodontic treatment. In general, mandibular molars have two roots with three or four canals [2]. However, three-rooted molars can occasionally be found, albeit with lower frequency. An additional third root is known as distolingual root, which is a normal anatomical variation in mandibular molars, especially mandibular first molars [3]. It was first mentioned by Carabelli as an extra root located at disto-lingual part of the tooth [4]. Several studies have used the term ‘Radix Entomolaris’ to describe it. The prevalence of distolingual roots is 3–10% worldwide [5–7]. There is a relationship between the frequency of distolingual roots and different ethnicities. Distolingual roots present more often in Mongoloid traits (more than 20%) than in Caucasian (0.7–4.2%) and Negroid traits (3.12%) [8–14]. In Thailand, the occurrence of distolingual roots ranges between 12.1-19.23% [15–17]. In endodontic therapy, clinicians occasionally fail to identify the existence of this supernumerary root because it can be superimposed by distal root in radiographic image. Furthermore, distolingual roots have hidden root canal curvature in the bucco-lingual plane which cannot be detected by conventional radiography [18–20]. Without awareness of this morphology, the hidden curve may cause complications such as ledging, perforation, and instrument separation during operation. Thus, persistent infection can occur due to missed canal and inadequate disinfection [21]. Since the occurrence of distolingual roots is associated with races, there have not been many studies observing its prevalence and morphology in the Thai population. Limited sample sizes and insufficient analysis of its morphology hinder the true understanding of this additional root. The purpose of this study is to investigate the prevalence and morphological characteristics of distolingual roots in permanent mandibular molars among a Thai population using a cone-beam computed tomography (CBCT).

## Methods

This retrospective study was approved by the Ethical Review Committee for Human Research Office of the Faculty of Dentistry and Faculty of Pharmacy, Mahidol University, Bangkok, Thailand (MU-DT/PY-IRB 2021/009.2801).

CBCT images of permanent first and second molars were obtained from Thai patients at the Oral and Maxillofacial Radiology Clinic, Faculty of Dentistry, Mahidol University, Bangkok, Thailand from January 2013 to December 2020. Images were captured using a 3D Accuitomo 170 machine (J. Morita, Kyoto, Japan). The tube voltage and current were set at 90 kV and 5 mA, respectively, with an exposure time of 17.5 s. The voxel sizes were 0.125, 0.16, 0.25 mm and the field of view options were 6 × 6, 8 × 8, 10 × 10, 14 × 10 cm. One Volume Viewer software (J. Morita, Kyoto, Japan) analyzed CBCT images in three standard planes (axial, coronal, and sagittal) with a slice thickness of 1 mm.

An examiner calibrated with a Thai-board certificated endodontist to verify inter-rater reliability before evaluation. Inter-rater and intra-rater reliability were assessed using Intraclass correlation coefficient (ICC) and Cohen’s Kappa, which showed almost perfect agreement (ICC = 0.93, Cohen’s Kappa = 0.97). The study divided into two main parts: a prevalence study and a morphology study. After data collection, statistical analysis was performed.

### Part I: prevalence study

The inclusion criteria comprised CBCT images of Thai patients whose permanent mandibular first or second molars could be detected. The exclusion criteria were as follows: (1) CBCT images with intrabony lesions or artifacts obstructing interpretation. (2) Teeth with root resorption exceeding two-thirds of the entire root length.

The presence of distolingual root was recorded when it was clearly separate from distal root. Additional data, including gender, tooth type, and topologic predilection, were collected. For mandibular first molars with distolingual root, the prevalence of unusual morphology in adjacent mandibular first and second premolars was collected. Data on bilateral concurrence was collected if the entire mandibular arch could be interpreted in the patients with bilateral mandibular first or second molars.

Descriptive statistics were presented as frequencies or percentages. Pearson’s chi-square test analyzed the presence of distolingual roots according to gender, whereas McNemar’s test was used for tooth type and topologic predilection. Statistical significance was set at p-value ≤ 0.05, and IBM SPSS Statistics for Windows, version 26 (IBM Corp., Armonk, NY, USA), was used to performed statistical analysis.

### Part II: morphology study

The Inclusion criteria encompassed CBCT images of Thai patients detecting distolingual roots of permanent mandibular first or second molars with field of view options of 6 × 6 cm or 8 × 8 cm and voxel sizes of 0.125–0.16 mm. The exclusion criteria were as follows: (1) CBCT images with intrabony lesion or artifacts hindering interpretation. (2) Teeth with incomplete root formation, root resorption, calcified pulp chamber, prior therapy, or unclear visualization due to image quality.

Prior to evaluation, to mitigate potential biases in image plane interpretation, all CBCT images were standardized by aligning the cementoenamel junction (CEJ) at the mesial and distal aspects of the crown to ensure consistency in the vertical plane across all images.

Distolingual root types were classified using Carlsen and Alexandersen’s [22] and Song’s [23] classification, whereas Vertucci’s [24] classification criteria were applied for root canal configuration. Interorifice distances between distobuccal-distolingual (DB-DL), mesiolingual-distolingual (ML-DL), and mesiobuccal-mesiolingual (MB-ML) orifices were measured at the starting point of DB-DL orifices separation in the axial view. The mean angle formed by the distobuccal, distolingual, and mesiolingual (DB-DL-ML) orifices was determined, and the vertical distance from the pulpal floor to the starting point of distolingual orifice separation was measured in the clinical view. The angle and radius of curvature were analyzed using Schneider’s [25] and Weine’s [26] methods and Pruett’s [27] method, respectively, in the clinical and proximal views.

Descriptive statistics were presented for root types and canal configuration, while the mean values and standard deviations for other measurements. Paired t-test and Wilcoxon signed-rank test were used to compared the mean differences in the angle and radius of curvature between the clinical and proximal views. Statistical significance was set at p-value ≤ 0.05, analyzed using IBM SPSS Statistics for Windows, version 26.

## Results

### Part I: prevalence study

**Fig. 1:**
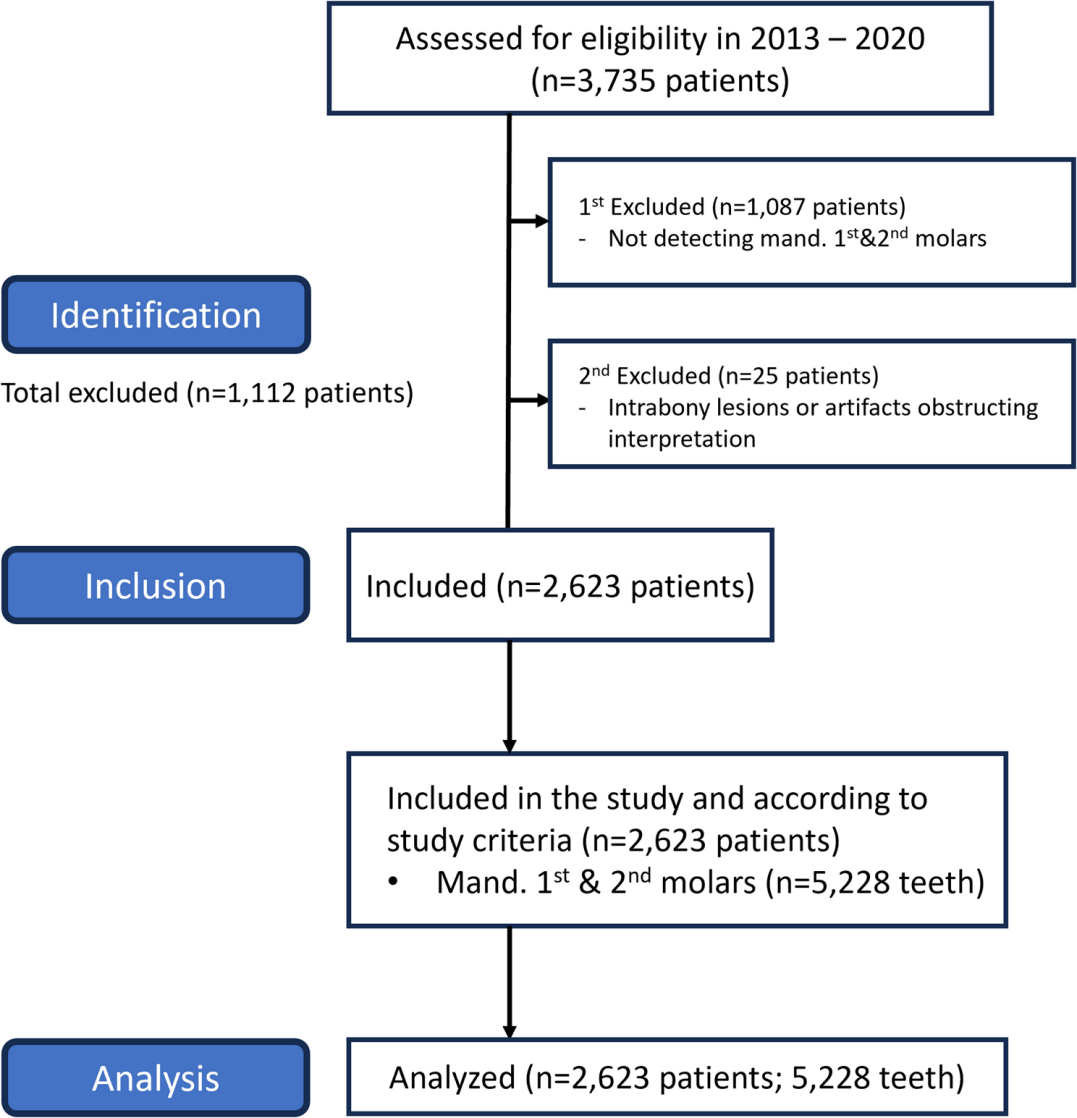
STROBE flow chart. STROBE, strengthening the reporting of observational studies in epidemiology

According to the inclusion and exclusion criteria, a total of 5228 permanent mandibular first and second molars were included in this study (Fig. 1). These comprised 2447 mandibular first molars and 2781 mandibular second molars from 2623 Thai patients, including 845 males and 1778 females, with a mean age of 42 years (range 18–65 years).

CBCT images revealed that the total prevalence of distolingual roots in permanent mandibular molars was 9.14% (478 out of 5228 examined teeth; 95% CI: 8.39–9.95%) and 14.94% (392 out of 2623 examined patients; 95% CI: 13.58–16.31%). The prevalence of distolingual roots in first mandibular molars was 18.43% (451 out of 2447 examined teeth; 95% CI: 16.94–20.02%) and 19.49% (376 out of 1929 examined patients; 95% CI: 17.79–21.32%) among the patients. In contrast, the presence of distolingual roots in second mandibular molars was significantly lower, at only 0.97% (27 out of 2.781 examined teeth; 95% CI: 0.67–1.41%) and 1.1% (24 out of 2169 examined patients; 95% CI: 0.74–1.64%). This finding indicates a statistically significant difference compared with the prevalence observed in first mandibular molars (*p* < 0.001). The prevalence of distolingual roots in males and females was 17.86% (151 out of 845 males; 95% CI: 15.43–20.60%) and 13.55% (241 out of 1778 females; 95% CI: 12.04–15.22%), respectively. When considering gender, males presented a greater occurrence of distolingual roots than females (*p* = 0.004; OR = 1.39, 95% CI: 1.11–1.73).

Additionally, the prevalence of unusual morphology in permanent mandibular premolars was recorded in patients with mandibular first molars with distolingual root. A total of 273 mandibular first premolars, 326 mandibular second premolars were examined. Among these cases, the prevalence of unusual morphology in mandibular first premolars was 39.56% (108 out of 273 examined teeth; 95% CI: 33.94– 45.47%; two roots 5 cases, three roots 2 cases, bifurcation 29 cases, trifurcation 6 cases, c-shaped 61 cases, Vertucci type III 5 cases). The prevalence of unusual morphology in mandibular second premolars was 4.6% (15 out of 326 examined teeth; 95% CI: 2.81– 7.45%; three roots 2 cases, bifurcation 6 cases, c-shaped 5 cases, Vertucci type III 2 cases).

A total of 737 CBCT images showing bilateral permanent first and/or second molars were further analyzed. These images comprised 518 bilateral mandibular first molars and 612 bilateral mandibular second molars, obtained from 250 male patients and 487 female patients. The prevalence of bilateral concurrence of distolingual roots was 10.58% (78 out of 737 patients; 95% CI: 8.56– 13.01%). No statistically significant difference was found between genders. Furthermore, in the unilateral group, a greater prevalence of distolingual roots occurred on the right side of mandibular first molars than on the left side (*p* = 0.002). However, this study found no statistically significant differences in the prevalence of distolingual roots between the two sides of the arch in mandibular second molars.

### Part II: morphology study

According to the inclusion and exclusion criteria, a total of 288 permanent mandibular first and second molars were included in this study. The root type of distolingual roots was categorized using Carlsen and Alexandersen’s classification and Song’s classification. According to Carlsen and Alexandersen’s classification, type C was the most prevalent, followed by type A, type B, and a single case of type AC, as shown in Table 1.

**Table 1:** Number and percentage of distolingual roots in permanent mandibular first and second molars according to Carlsen and Alexandersen’s classification

Tooth type	A (%)	B (%)	C (%)	AC (%)
Mandibular first molars (*n* = 277)	2 (0.7)	3 (1.1)	271 (97.8)	1 (0.4)
Mandibular second molars (*n* = 11)	2 (0.2)	0 (0)	9 (81.8)	0 (0)
Total (*n* = 288)	4 (1.4)	3 (1)	280 (97.2)	1 (0.3)

For Song’s classification, a total of 236 out of 288 teeth from the previously mentioned section were analyzed. A total of 52 teeth were excluded from this part of the study because the entire root was not completely included in CBCT images or some part of the root canal could not be seen. Type III was the most common, followed by type II, with only one case each for type I and small type. None of the teeth fell into conical type category, as shown in Table 2.

**Table 2:** Number and percentage of distolingual roots in permanent mandibular first and second molars according to song’s classification

Tooth type	I (%)	II (%)	III (%)	Small (%)	Conical (%)
Mandibular first molars (*n* = 225)	1 (0.4)	19 (8.4)	204 (90.7)	1 (0.4)	0 (0)
Mandibular second molars (*n* = 11)	0 (0)	2 (18.2)	9 (81.8)	0 (0)	0 (0)
Total (*n* = 236)	1 (0.4)	21 (8.9)	213 (90.3)	1 (0.4)	0 (0)

Additionally, Vertucci’s classification criteria was used to classify root canal configuration. The vast majority of the teeth (99.15%) were classified as type I. Notably, two permanent mandibular first molars were type III.

**Fig. 2:**
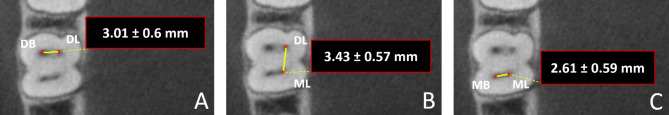
The axial view of the pulpal floor showing the mean distance (± standard deviation) in millimeters between (**A**) DB-DL orifices, (**B**) ML-DL orifices, and (**C**) MB-ML orifices. DB: distobuccal orifice. DL: distolingual orifice. MB: mesiobuccal orifice. ML: mesiolingual orifice

**Fig. 3:**
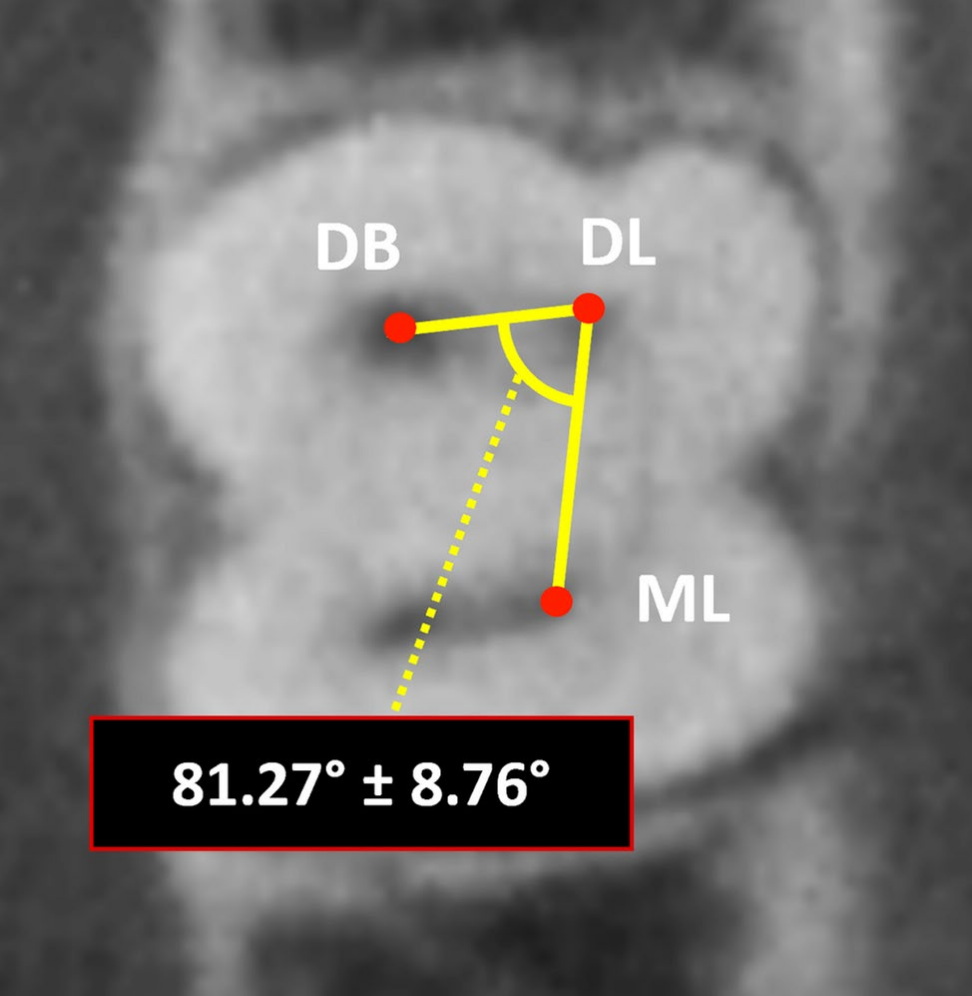
The axial view of the pulpal floor showing the mean angle (± standard deviation) in degree of DB-DL-ML orifices. DB: distobuccal orifice. DL: distolingual orifice. ML: mesiolingual orifice

For interorifice distances between DB-DL, ML-DL, and MB-ML orifices, 186 out of 236 teeth from the previously mentioned section were analyzed. However, 50 teeth were excluded for various reasons, including the inability to observe one or both sides of the cemento-enamel junction (CEJ), the presence of a calcified pulp chamber, and the mesial root having only one orifice. The mean distance between DB-DL orifices was measured to be 3.01 ± 0.6 mm. The mean distance between ML-DL orifices was 3.43 ± 0.57 mm, and the mean distance between MB-ML orifices was 2.61 ± 0.59 mm (Fig. 2). The angle of DB-DL-ML orifices was measured to be 81.27° ± 8.76° (Fig. 3). The mean vertical distance from the pulpal floor to the starting point where distolingual orifices were separated from distobuccal orifices was measured to be 1.51 ± 0.64 mm.

For the angle and radius of curvature of distolingual roots, 123 out of 186 teeth from the previously mentioned section were analyzed. A total of 63 teeth were excluded because of severe curvatures and multiple curves in the root canal, making it impossible to measure the entire root canal on one slide. In the clinical view, the majority of cases were categorized as severely curved and as having a small radius. Four cases were unable to be measured because of a straight canal with a 0° angle of curvature.

In the proximal view, all cases were categorized as severely curved and the majority were classified as having a small radius as shown in Table 3.

**Table 3:** Number of distolingual roots in each category of curvature

View	Angle of curvature (Schneider’s method)			Radius of curvature		
	Straight (< 10°) (%)	Moderate (10° − 25°) (%)	Severe (> 25°) (%)	Small (< 4 mm) (%)	Moderate (4–8 mm) (%)	Large (> 8 mm) (%)
Clinical	6 (4.9)	11 (8.9)	106 (86.2)	54 (45.4)	43 (36.1)	22 (18.5)
Proximal	0 (0)	0 (0)	123 (100)	97 (78.9)	26 (21.1)	0 (0)

Statistically significant differences were observed when comparing the clinical and proximal views in both the angle of curvature (using Schneider’s and Weine’s methods) and the radius of curvature. The angle and radius of curvature in the proximal plane were greater than those in the clinical plane (*p* < 0.001) as shown in Table 4.

**Table 4:** The angle and radius of curvature in the clinical and proximal views

Technique	View		*P*-value
	Clinical	Proximal	
Angle of curvature (Schneider’s method)	22.04° ± 12.5°	45.39° ± 13.43°	*p* < 0.001*
Angle of curvature (Weine’s method)	29.69° ± 16.1°	63.61° ± 19.89°	*p* < 0.001*
Radius of curvature	6.07 ± 7.34 mm	3 ± 1.48 mm	*p* < 0.001**

* Analyzed by Paired T-test

** Analyzed by Wilcoxon signed-rank test

## Discussion

In this study, CBCT was utilized to investigate the prevalence and morphology of distolingual roots. Compared with conventional radiography, this nondestructive imaging technique enables the visualization of root or root canal systems in three dimensions without superimposition or distortion, revealing a greater occurrence of additional root [28]. Moreover, CBCT is as accurate as the modified canal staining method in investigating root canal systems [29].

Since this study utilized CBCT to analyze distolingual roots, it is essential to consider the ALARA (As Low As Reasonably Achievable) principle, which aims to minimize radiation exposure while ensuring sufficient diagnostic information. The ALARA principle is particularly important in CBCT studies because CBCT imaging generally delivers a higher radiation dose compared to conventional two-dimensional radiographs, such as periapical and panoramic images. Therefore, CBCT should only be used when there is a clear clinical indication and when alternative imaging modalities are insufficient. Additionally, parameters such as field of view, kVp, mA, and voxel size should be optimized to achieve the necessary image quality while minimizing radiation exposure [30]. 

The sample size in this study comprised a total of 5228 permanent mandibular molars collected from 2623 Thai patients, which is notably larger than that in previous studies. Previous studies reported sample sizes ranging from 105 to 1958 examined teeth in permanent mandibular molars and 86 to 558 examined patients [3, 10, 31, 32]. The substantial increase in sample size enhances the reliability of the results obtained from this study and brings them into closer alignment with the population characteristics.

This study revealed that the prevalence of distolingual roots in permanent mandibular first molars and second molars was 14.94% among individuals, which is similar to the findings of Reichart & Metah (19.2%) in the Thai population, but slightly greater than those of Gulabivala et al. (12.7%) and Hiran-us et al. (12.1%) [15–17]. While the prevalence of Mongolian traits has been reported to range from 8.5 to 33.3%, which is higher than that observed in Caucasian (0.7-4.2%) and Negroid traits (3.2%), these differences may not solely be attributed to racial variation but also to differences in sample sizes, inclusion criteria, imaging techniques, and classification methods [8, 9, 11, 33–36]. Notably, the greater prevalence of distolingual roots in mandibular first molars is consistent with the findings of previous studies [3, 37]. While most studies reported no gender association with the presence of distolingual roots, this study revealed a greater prevalence in males, which is consistent with the finding of Huang et al. [3, 6, 13, 31, 38].

This study revealed a bilateral concurrence prevalence of 56.39%, which is consistent with previous studies conducted in Taiwan and Korea, which reported the prevalence rate ranging from 53.65 to 78.57% [31, 35, 38]. This underscores the importance for clinicians to be cognizant of the potential for bilateral concurrence of distolingual roots in individuals of Mongoloid descent. In the unilateral group, this study revealed a significantly greater prevalence of distolingual roots on the right side in mandibular first molars. This finding is consistent with several other studies [34, 35, 38–40]. However, some studies have reported a greater prevalence on the left side, whereas others reported no significant difference between the two sides of the arch [6, 32, 33].

In patients with distolingual roots in mandibular first molars, the prevalence of unusual morphology in mandibular first premolars was 39.59%, with over half exhibiting a c-shaped root canal configuration. Wu et al. reported a positive correlation between c-shaped mandibular first premolars and the presence of distolingual roots in mandibular first molars [38]. This information can be useful for clinicians to consider when performing endodontic treatment on mandibular premolars in patients with distolingual roots, as these anatomical variations may be more commonly encountered in these cases. However, due to limitations in data collection and the relatively small sample size of mandibular premolars, these findings should be regarded as preliminary observations rather than established correlations. Future research is necessary to further investigate the relationship between distolingual roots in mandibular molars and anatomical variations in mandibular premolars to provide more robust evidence.

In morphological analysis, more than 90% of cases with distolingual roots were classified as type C according to Carlsen and Alexandersen’s classification. This finding suggests that distolingual root is located mainly mesially to distal root, particularly in the coronal one-third. Furthermore, only one case was categorized as type AC, defined as the coronal one-third of distolingual root located between mesial and distal roots, and distinguishable on conventional radiography without superimposition with distal root. Due to the limited number of type AC cases, several distolingual roots may be missed during interpretation using conventional radiography. In this study, the majority of cases (90.25%) were type III according to Song’s classification, which is consistent with the findings of other studies [10, 41]. In contrast, Song et al. reported the most prevalent type as type II (47.5%), followed by type III (40.5%) [23]. These discrepancies in results could be attributed to racial differences and variations in sample sizes among the studies. The results of this study also indicated that the majority of distolingual roots exhibited curvature in bucco-lingual plane.

According to Vertucci’s classification, 99.15% of distolingual roots in this study were categorized as type I, with the interesting finding that two distolingual roots fell under type III. Similarly, Rodrigues et al. also observed two distolingual roots that did not fall into type I, with configurations categorized as type V and VII [42]. This highlights the potential variability in the canal configuration of distolingual roots, which clinicians should be aware of.

Finding the distolingual orifice is challenging for clinicians and can easily be missed without proper awareness. This study provides valuable insight into the vertical positioning of distolingual orifices, revealing that they were separated from distobuccal orifices by approximately 1.51 ± 0.64 mm from the pulpal floor. This is the first investigation of its kind, aiding clinicians in accurately locating distolingual orifice within the vertical plane. Additionally, the mean distances between DB-DL and ML-DL orifices were 3.01 ± 0.6 mm and 3.43 ± 0.57 mm, respectively, which is consistent with previous studies. The mean MB-ML orifice distance was 2.61 ± 0.59 mm, which was slightly less than DB-DL orifices distance, which aligns with prior research [10, 20, 35, 42, 43]. Moreover, the mean angle formed by DB-DL-ML orifices was 81.27° ± 8.76°, indicating that distolingual orifices are located more disto-lingually from distobuccal orifices. These findings suggest that access cavity preparation should be modified to a more trapezoidal shape, extending distolingually, rather than a conventional triangular design to improve visualization and instrumentation of distolingual canals, reducing the risk of missed canal. Using interorifice distances to assess the relationship of distolingual orifice with adjacent orifices, along with considering its vertical distance from the pulpal floor, enhances the ability to locate it successfully.

In the analysis of the angle of curvature in the clinical view, the mean angle of curvature was 22.04° ± 12.5°, which was greater than that reported in other studies [20, 44]. The majority of cases exhibited severe curvature, which contrasts with findings from other studies reporting a higher prevalence of cases with moderately curved or straight canal [19, 20]. This discrepancy may arise from differences in the evaluation methods utilized, highlighting the need for further investigation and standardization in assessing root canal curvatures.

In the analysis of the angle of curvature in the proximal view, the mean angle of curvature measured 45.39° ± 13.43°, closely resembling the findings of several studies where the mean angle ranged between 45.39° and 59.04° [19, 42]. Furthermore, all cases in this study exhibited a severely curved canal, which is consistent with other studies reporting a higher prevalence of severely curved canals [19, 20].

When comparing the clinical and proximal views, the angle of curvature in the proximal view was significantly greater, aligning with previous studies [18, 20].

In the radius of curvature analysis within the clinical view, the mean radius of curvature was 6.07 mm ± 7.34 mm, which was smaller than the findings of Gu et al. who reported a mean radius of 20.99 ± 19.35 mm. Similarly, in the radius of curvature analysis within the proximal view, the mean radius of curvature was 3 mm ± 1.48 mm, which was smaller than that reported by Gu et al. who reported a mean radius of 6.17 ± 2.51 mm. When comparing the clinical and proximal views, it was observed that the radius of curvature in the proximal view was statistically significantly lower, indicating a more abrupt curvature. These results are consistent with the findings of other studies [19, 45].

Importantly, out of the total 186 cases, approximately 1/3 were excluded from the angle and radius of curvature analysis due to severe or multiple curvature that could not be measured within a single slide of CBCT images. As a result, the findings of this study may underestimate the actual canal curvature of distolingual roots. These findings emphasize the importance of clinicians being mindful of root curvature to avoid potential procedural errors. Heat-treated or flexible NiTi rotary files with reduced taper, pre-curved stainless steel hand files, and a glide path with small K-files (#08 or #10) help navigate sharp curves while minimizing procedural errors. Passive irrigation and frequent recapitulation improve canal negotiation and debris removal, preserving canal integrity and reducing complications such as ledging or instrument fracture. These techniques enhance the success of endodontic treatment in distolingual roots [46, 47].

Despite the valuable insights gained from this study, certain limitations must be acknowledged. First, the study utilized CBCT images from a single population, which may limit the generalizability of the findings to other demographic groups. Additionally, the CBCT technology employed in this study, which primarily acquires images in two-dimensional slices rather than a fully reconstructed three-dimensional format, limited the ability to assess severe or multiple root curvatures. Future studies involving larger, more diverse populations and standardized imaging protocols are recommended to further validate these findings and enhance the understanding of distolingual root variations.

## Conclusions

This study found the prevalence of distolingual roots in permanent mandibular molars to be 14.94%, with a higher occurrence in first molars. Most distolingual roots were classified as type C according to Carlsen and Alexandersen’s classification and type III according to Song’s classification, with the majority exhibiting a Vertucci type I canal configuration. Distolingual orifice was typically located disto-lingually to distobuccal orifice, with an average separation of 1.5 ± 0.64 mm from the pulpal floor. Importantly, distolingual roots often exhibited severe and abrupt curvature, particularly in bucco-lingual plane, which may complicate endodontic procedures. These findings highlight the importance of understanding the intricacies of distolingual roots to enhance treatment outcomes and reduce procedural complications in clinical practice.

## Data Availability

The datasets used and/or analysed during the current study are available from the corresponding author on reasonable request.

## Abbreviations

CBCT: Cone beam computed tomography
CEJ: Cementoenamel junction
DB: Distobuccal
DL: Distolingual
MB: Mesiobuccal
ML: Mesiolingual
